# Supplementary material for: Absolute quantum yield measurements of fluorescent proteins using a plasmonic nanocavity
Source: Commun Biol. 2020 Oct 30;3:627. doi: 10.1038/s42003-020-01316-2 (PMC7599333; doi:10.1038/s42003-020-01316-2)
Supplement: Supplementary file 1 — Supplementary Information [file 42003_2020_1316_MOESM1_ESM.pdf]

Supplementary Information

Absolute quantum yield measurements of fluorescent proteins using a plasmonic nanocavity

Daja Ruhlandt[1], Martin Andresen[2], Nickels Jensen[2], Ingo Gregor[1], Stefan Jakobs[2,3], Jörg Enderlein*[1], Alexey I. Chizhik*[1]

[1]Georg-August-University Göttingen, Third Institute of Physics - Biophysics, Göttingen, Germany

[2]Max Planck Institute for Biophysical Chemistry, Department of NanoBiophotonics, Am Fassberg 11, 37077 Göttingen, Germany

[3]University of Göttingen Medical Faculty, Department of Neurology, Robert-Koch-Strasse 40, 37075 Göttingen, Germany

* Corresponding authors:

J.E.: jenderl@gwdg.de

A.I.C.: alexey.chizhik@phys.uni-goettingen.de

Supplementary Figures

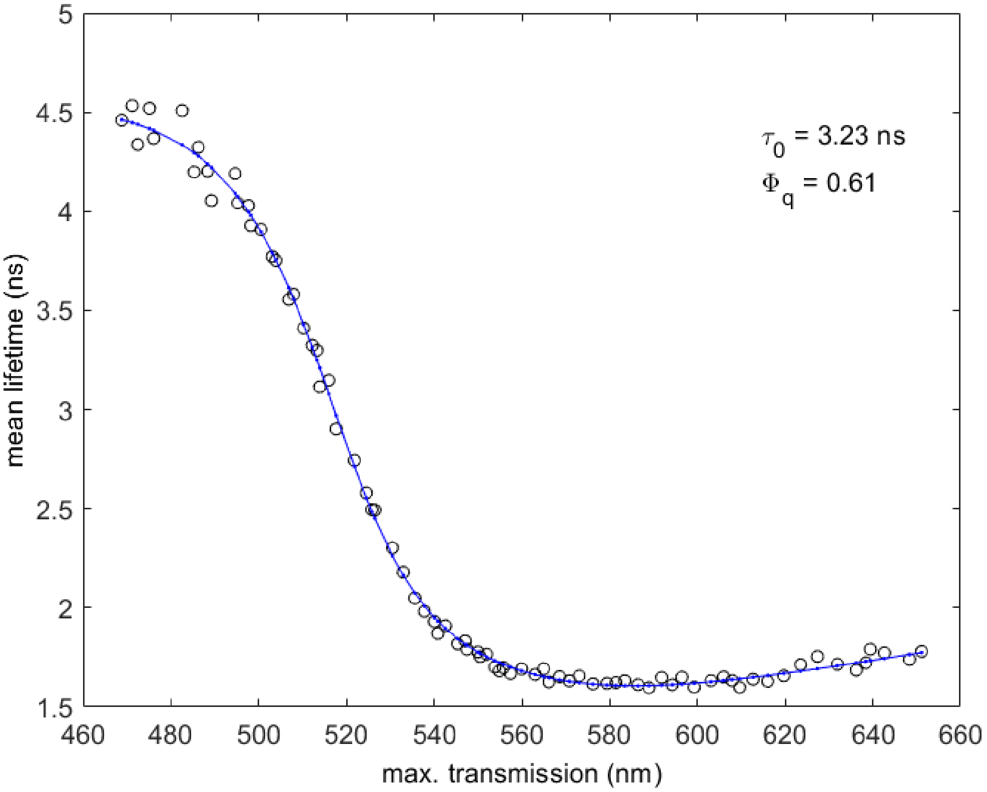

Supplementary Figure 1. (a) Excited state lifetime of Citrine as a function of the maximum transmission wavelength (linearly proportional to the cavity length) of the nanocavity. The fit parameters are the fluorescence quantum yield (Φ) and the free lifetime in the absence of the cavity (τ).

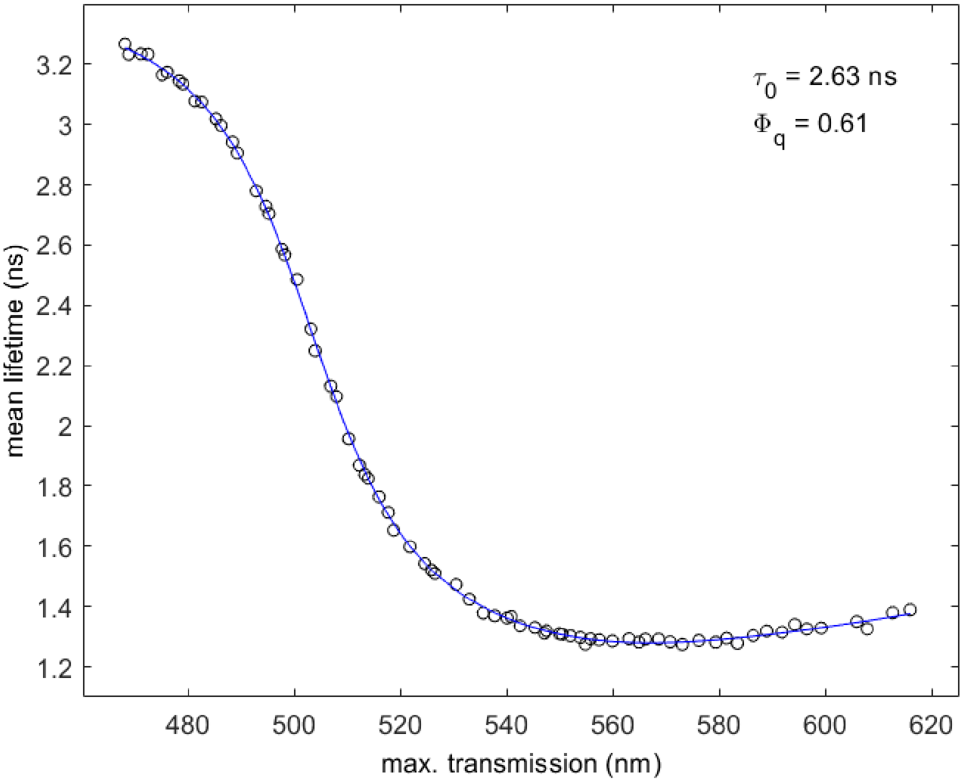

Supplementary Figure 2. (a) Excited state lifetime of EGFP as a function of the maximum transmission wavelength (linearly proportional to the cavity length) of the nanocavity. The fit parameters are the fluorescence quantum yield (Φ) and the free lifetime in the absence of the cavity (τ).

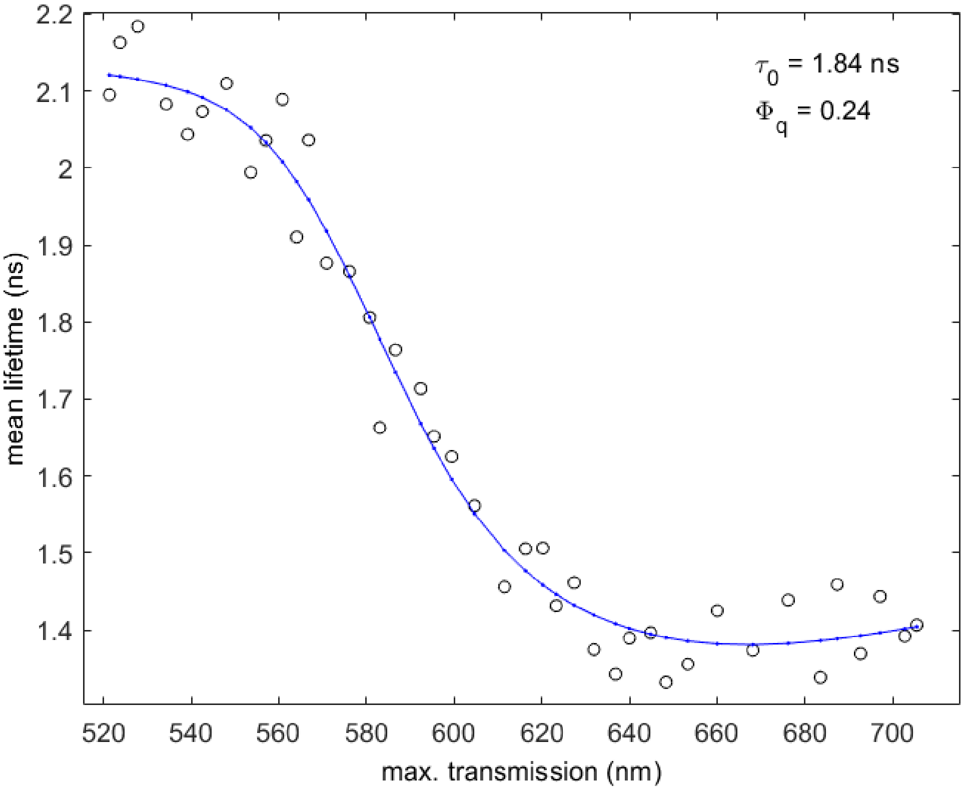

Supplementary Figure 3. (a) Excited state lifetime of mCherry as a function of the maximum transmission wavelength (linearly proportional to the cavity length) of the nanocavity. The fit parameters are the fluorescence quantum yield (Φ) and the free lifetime in the absence of the cavity (τ).

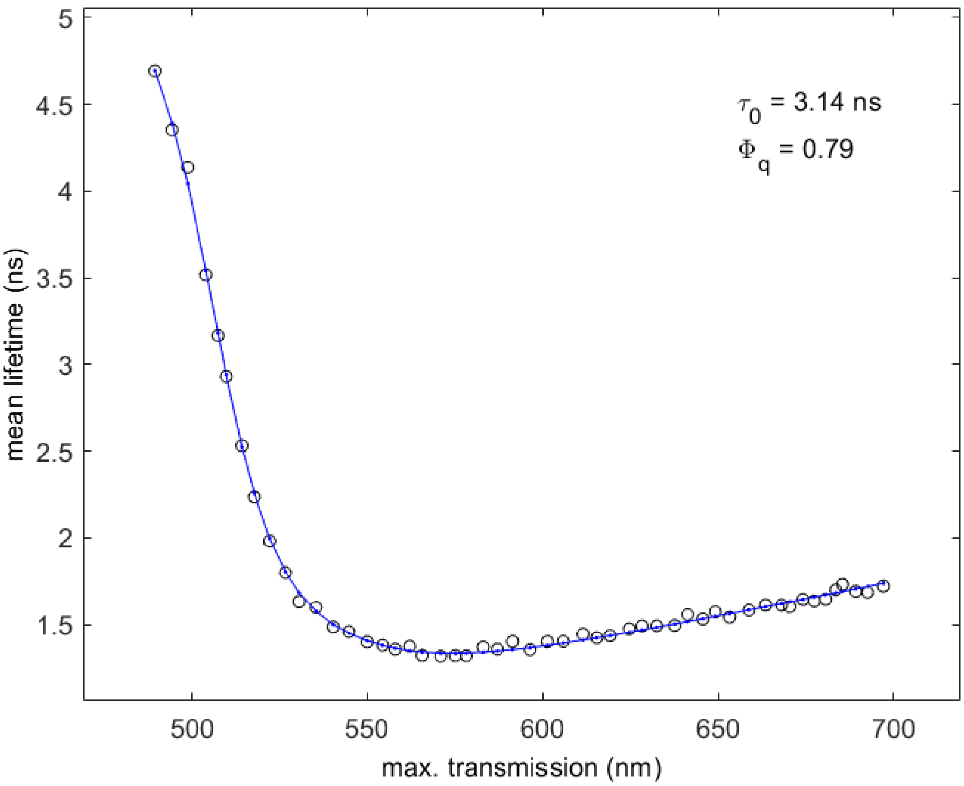

Supplementary Figure 4. (a) Excited state lifetime of Clover as a function of the maximum transmission wavelength (linearly proportional to the cavity length) of the nanocavity. The fit parameters are the fluorescence quantum yield (Φ) and the free lifetime in the absence of the cavity (τ).

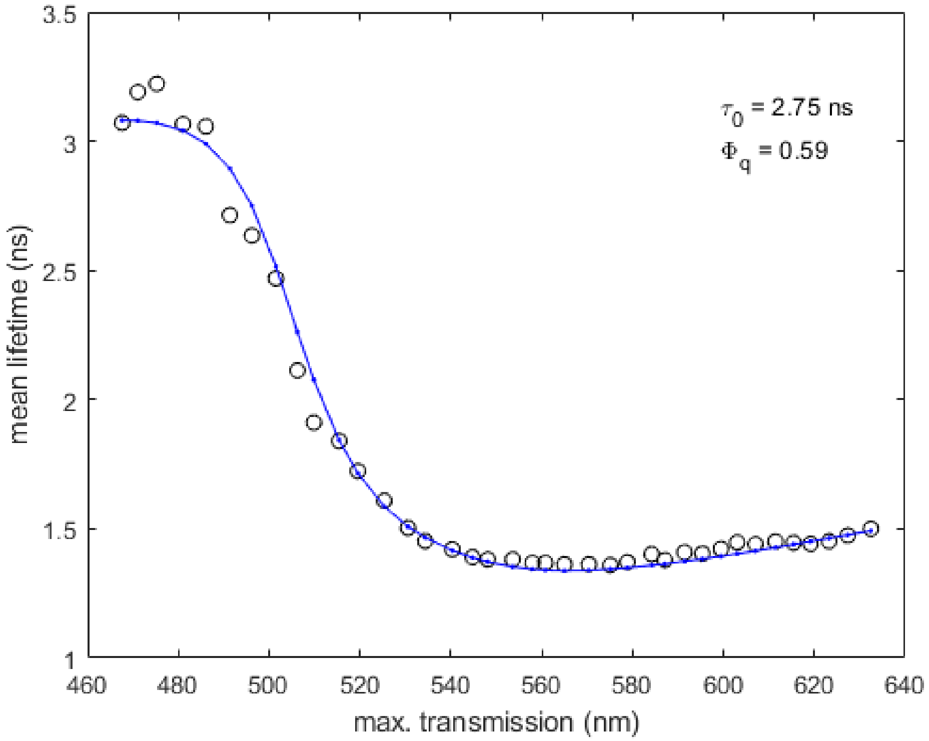

Supplementary Figure 5. (a) Excited state lifetime of mEGFP as a function of the maximum transmission wavelength (linearly proportional to the cavity length) of the nanocavity. The fit parameters are the fluorescence quantum yield (Φ) and the free lifetime in the absence of the cavity (τ).

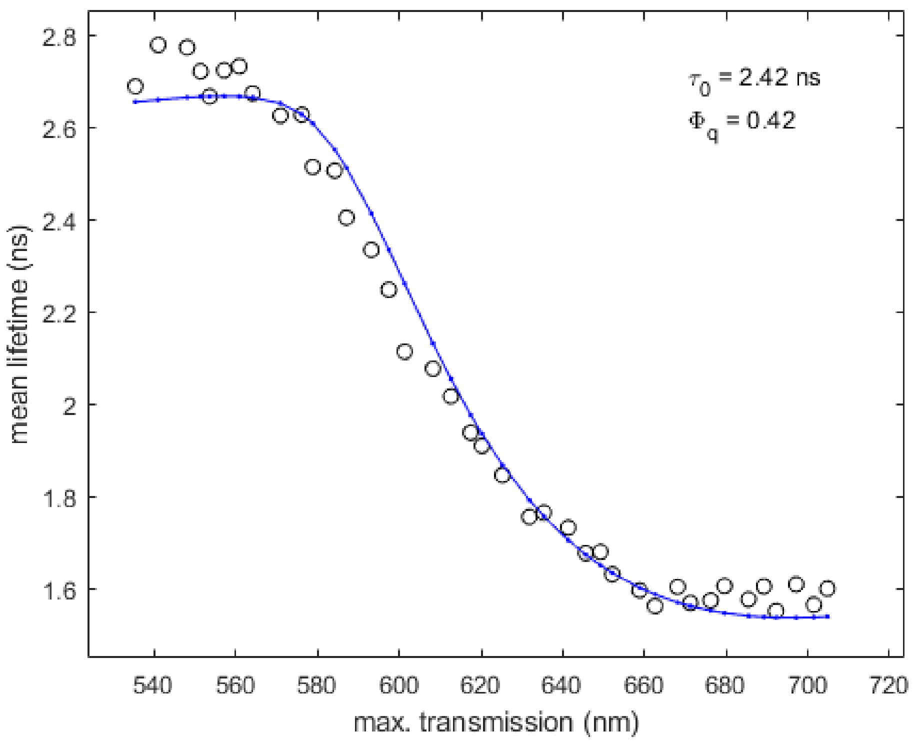

Supplementary Figure 6. (a) Excited state lifetime of mKate2 as a function of the maximum transmission wavelength (linearly proportional to the cavity length) of the nanocavity. The fit parameters are the fluorescence quantum yield (Φ) and the free lifetime in the absence of the cavity (τ).

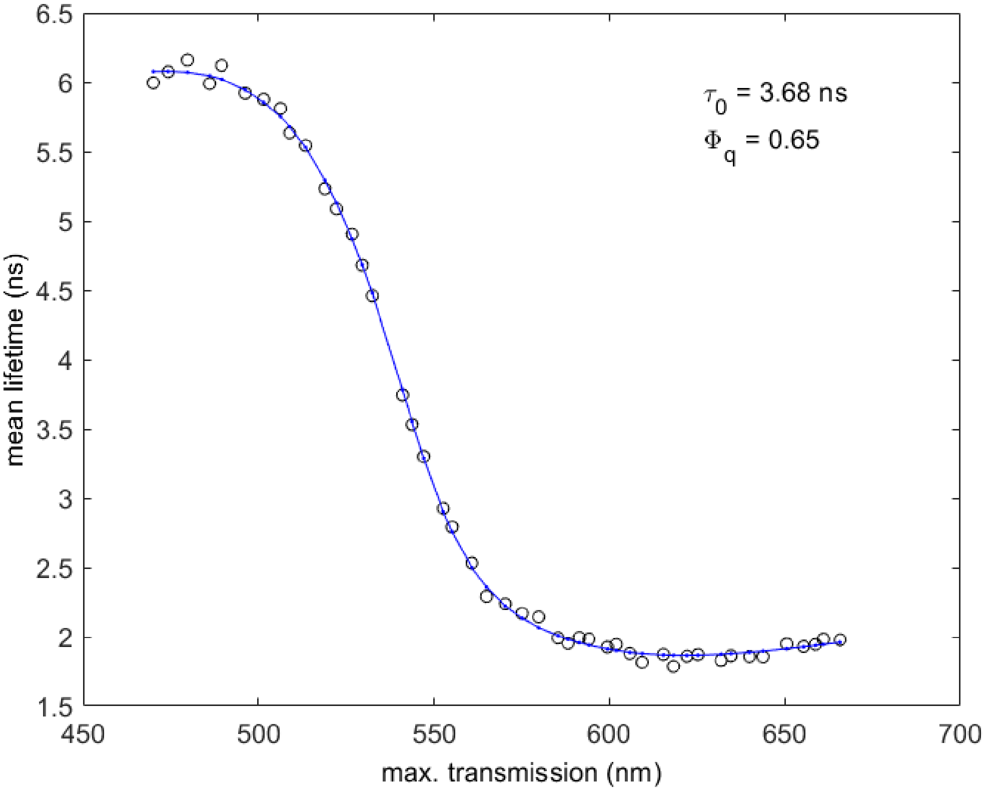

Supplementary Figure 7. (a) Excited state lifetime of mKO2 as a function of the maximum transmission wavelength (linearly proportional to the cavity length) of the nanocavity. The fit parameters are the fluorescence quantum yield (Φ) and the free lifetime in the absence of the cavity (τ).

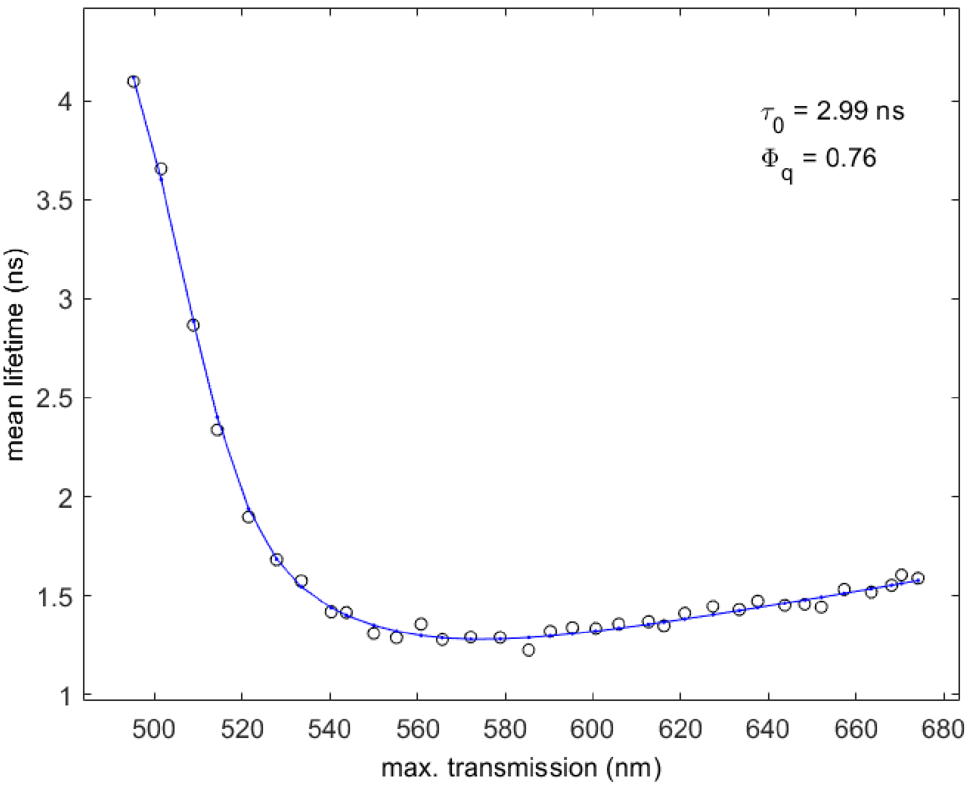

Supplementary Figure 8. (a) Excited state lifetime of mNeonGreen as a function of the maximum transmission wavelength (linearly proportional to the cavity length) of the nanocavity. The fit parameters are the fluorescence quantum yield (Φ) and the free lifetime in the absence of the cavity (τ).

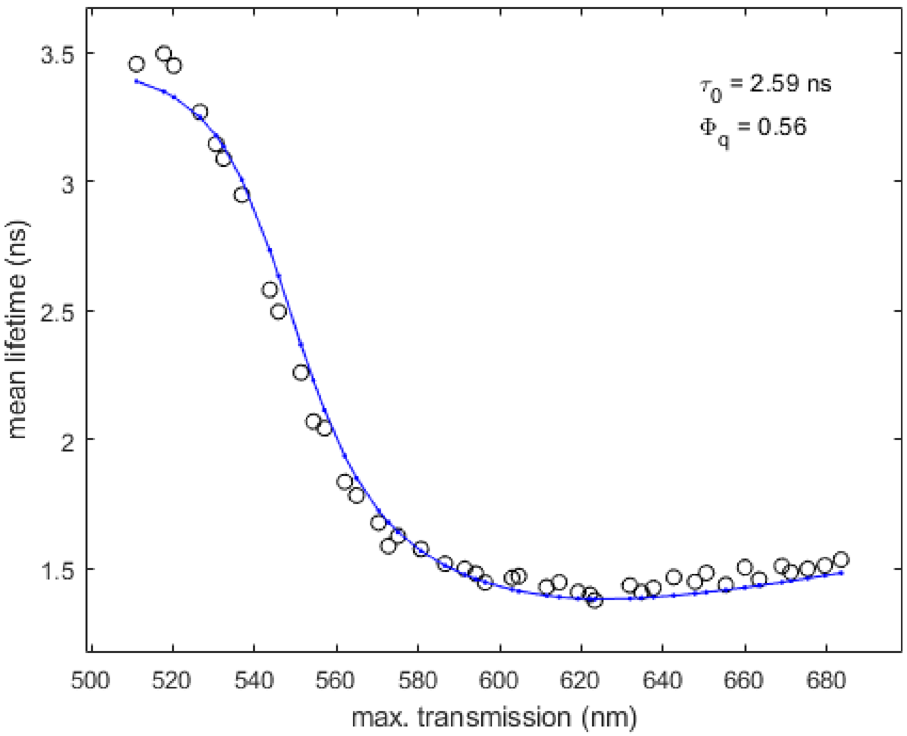

Supplementary Figure 9. (a) Excited state lifetime of mOrange2 as a function of the maximum transmission wavelength (linearly proportional to the cavity length) of the nanocavity. The fit parameters are the fluorescence quantum yield (Φ) and the free lifetime in the absence of the cavity (τ).

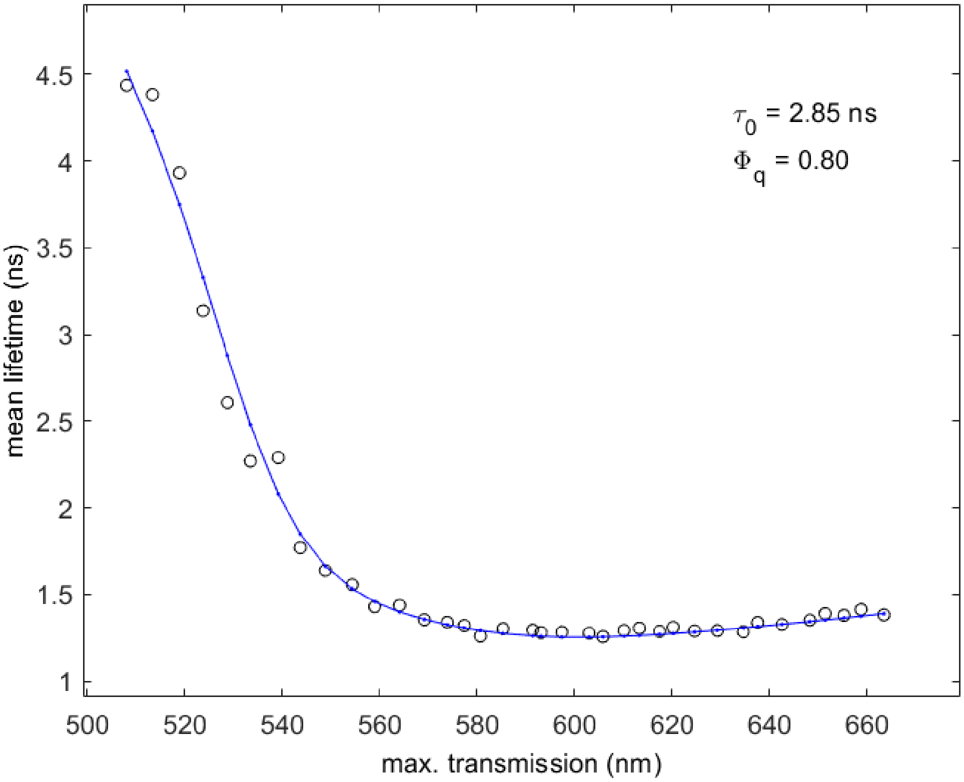

Supplementary Figure 10. (a) Excited state lifetime of mPapaya as a function of the maximum transmission wavelength (linearly proportional to the cavity length) of the nanocavity. The fit parameters are the fluorescence quantum yield (Φ) and the free lifetime in the absence of the cavity (τ).

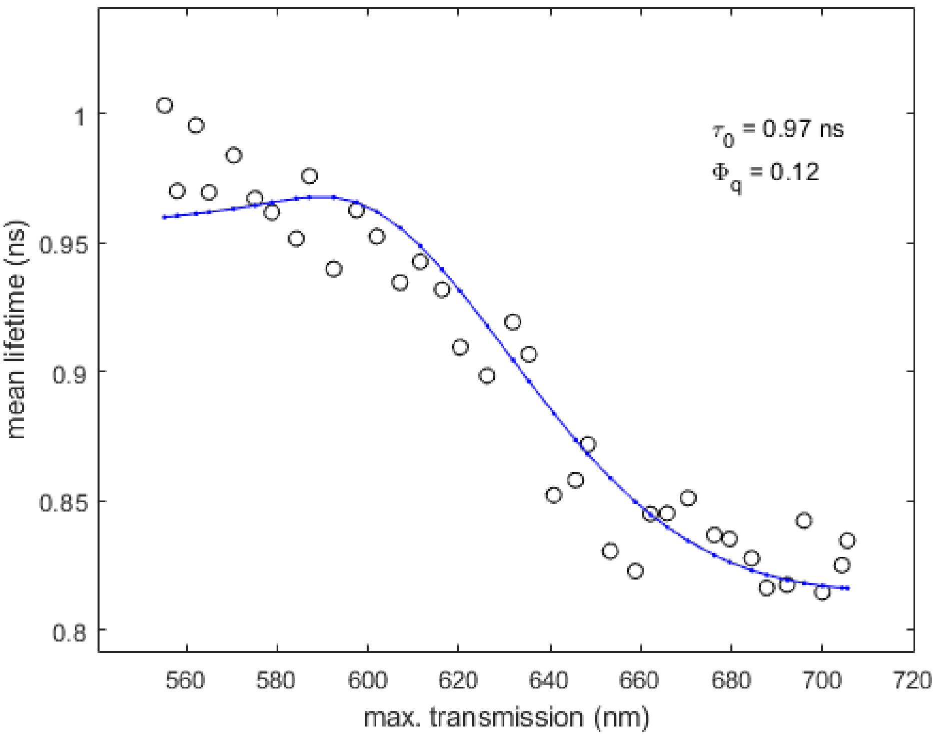

Supplementary Figure 11. (a) Excited state lifetime of mPlum as a function of the maximum transmission wavelength (linearly proportional to the cavity length) of the nanocavity. The fit parameters are the fluorescence quantum yield (Φ) and the free lifetime in the absence of the cavity (τ).

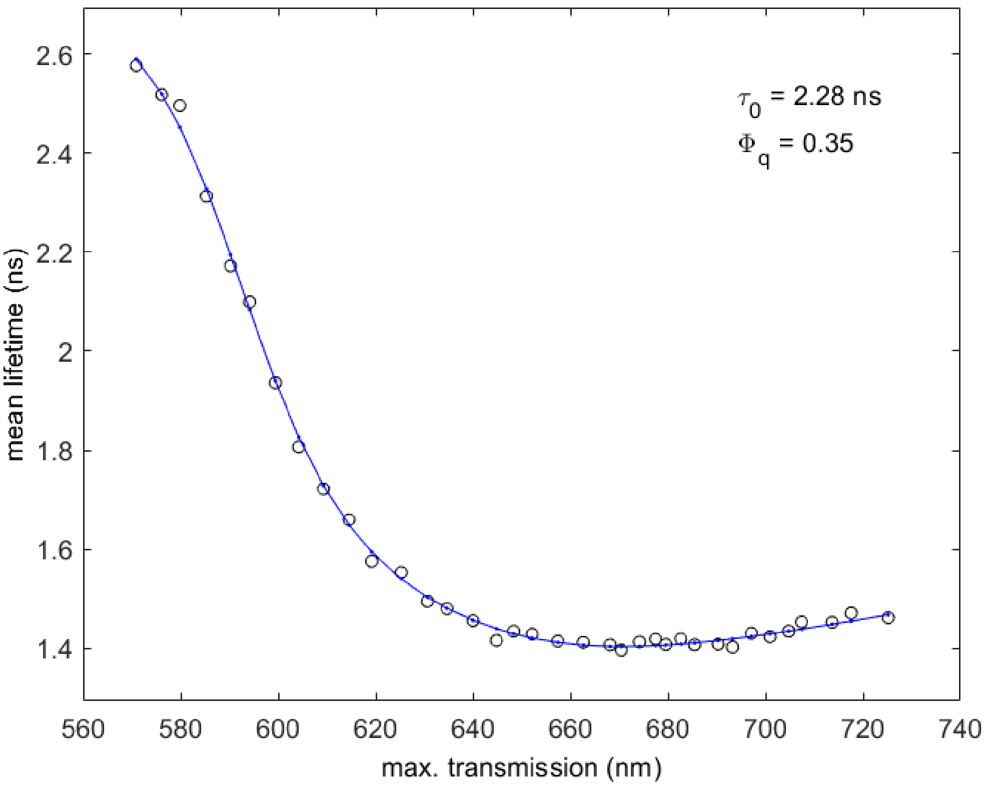

Supplementary Figure 12. (a) Excited state lifetime of mRuby2 as a function of the maximum transmission wavelength (linearly proportional to the cavity length) of the nanocavity. The fit parameters are the fluorescence quantum yield (Φ) and the free lifetime in the absence of the cavity (τ).

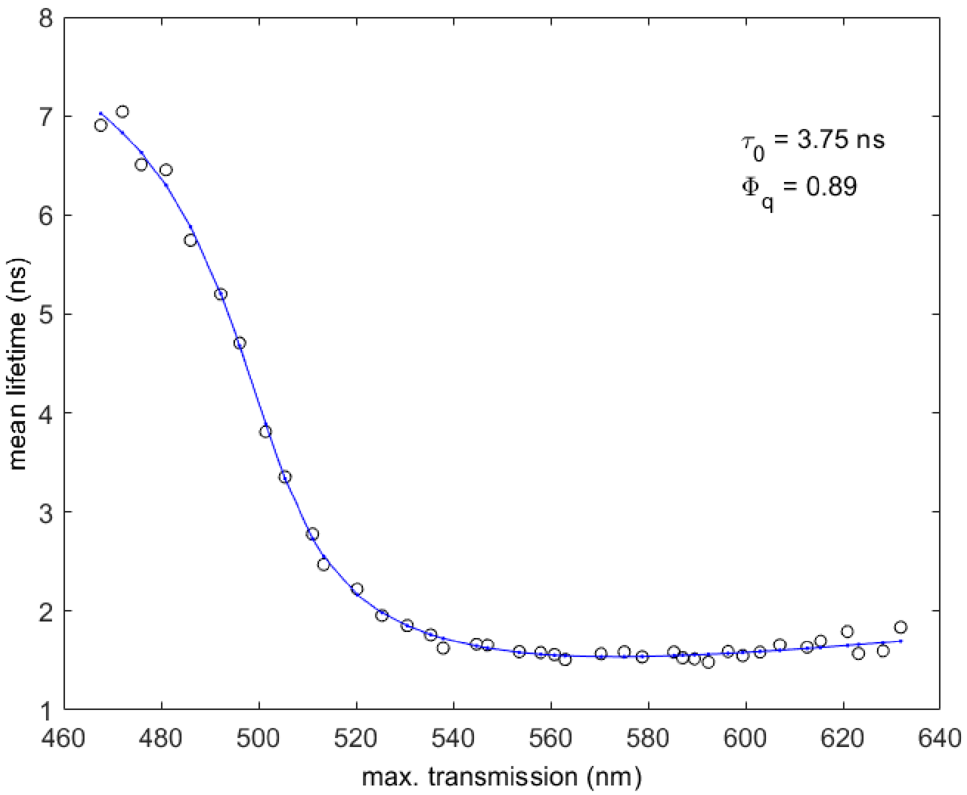

Supplementary Figure 13. (a) Excited state lifetime of mTurquoise2 as a function of the maximum transmission wavelength (linearly proportional to the cavity length) of the nanocavity. The fit parameters are the fluorescence quantum yield (Φ) and the free lifetime in the absence of the cavity (τ).

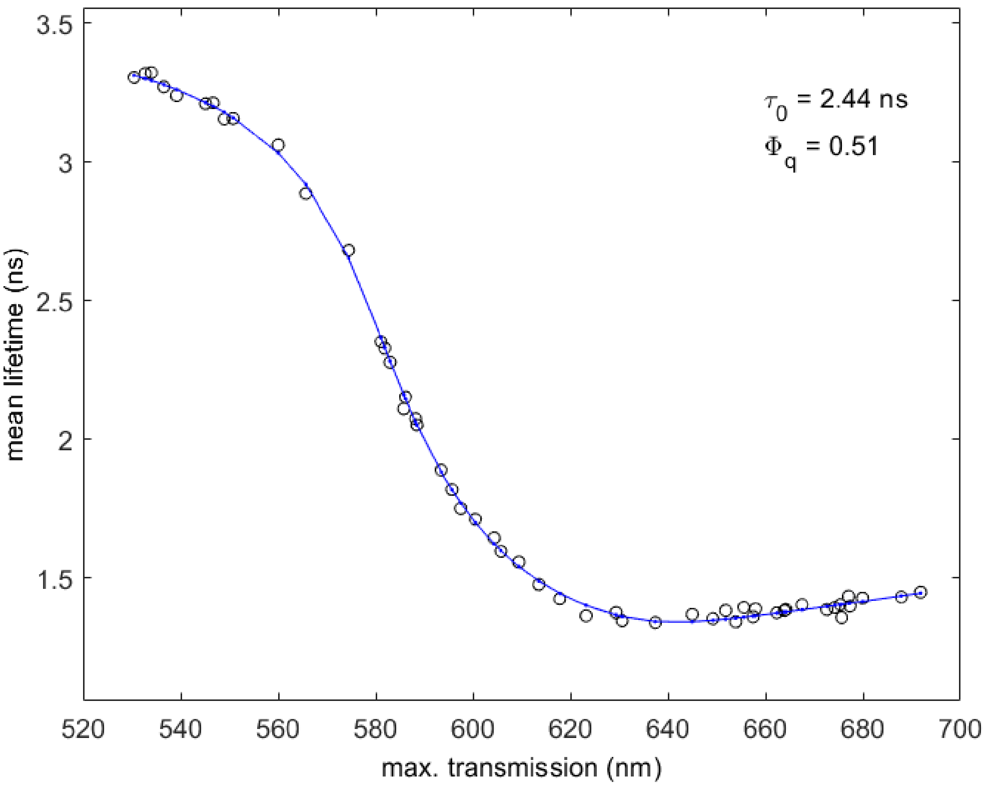

Supplementary Figure 14. (a) Excited state lifetime of TagRFP as a function of the maximum transmission wavelength (linearly proportional to the cavity length) of the nanocavity. The fit parameters are the fluorescence quantum yield (Φ) and the free lifetime in the absence of the cavity (τ).

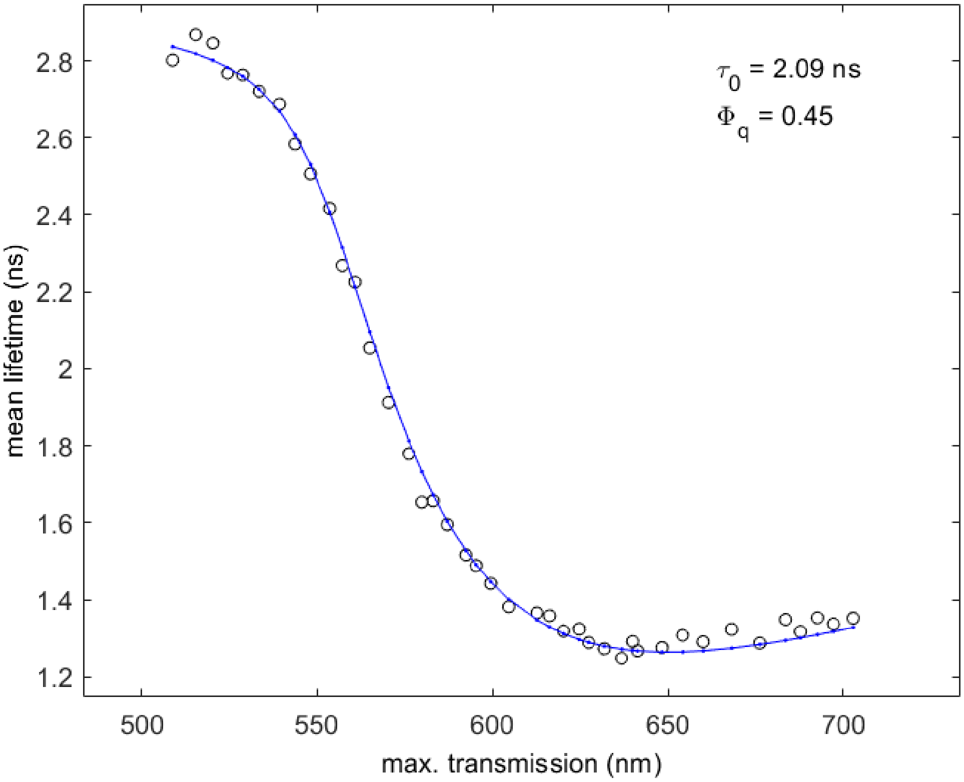

Supplementary Figure 15. (a) Excited state lifetime of TagRFP-T as a function of the maximum transmission wavelength (linearly proportional to the cavity length) of the nanocavity. The fit parameters are the fluorescence quantum yield (Φ) and the free lifetime in the absence of the cavity (τ).

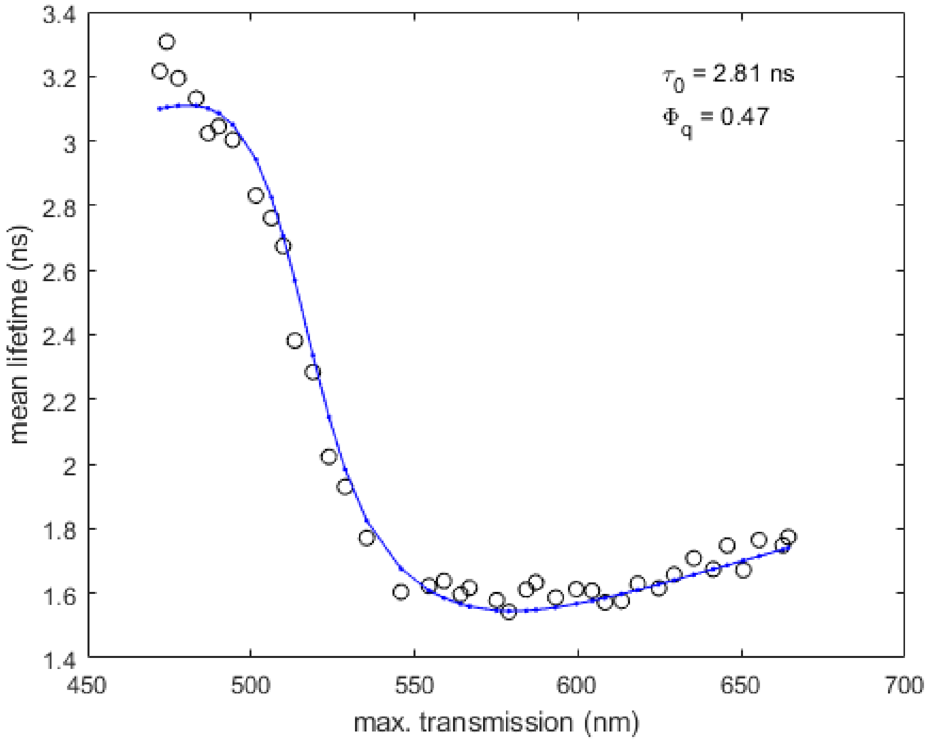

Supplementary Figure 16. (a) Excited state lifetime of Dreiklang as a function of the maximum transmission wavelength (linearly proportional to the cavity length) of the nanocavity. The fit parameters are the fluorescence quantum yield (Φ) and the free lifetime in the absence of the cavity (τ).

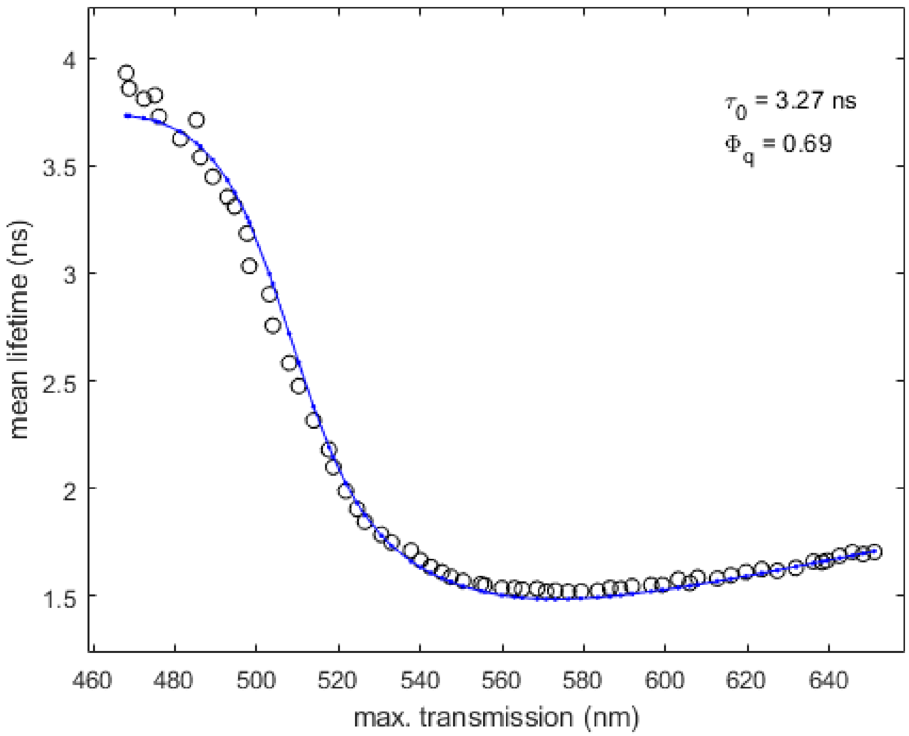

Supplementary Figure 17. (a) Excited state lifetime of Dronpa as a function of the maximum transmission wavelength (linearly proportional to the cavity length) of the nanocavity. The fit parameters are the fluorescence quantum yield (Φ) and the free lifetime in the absence of the cavity (τ).

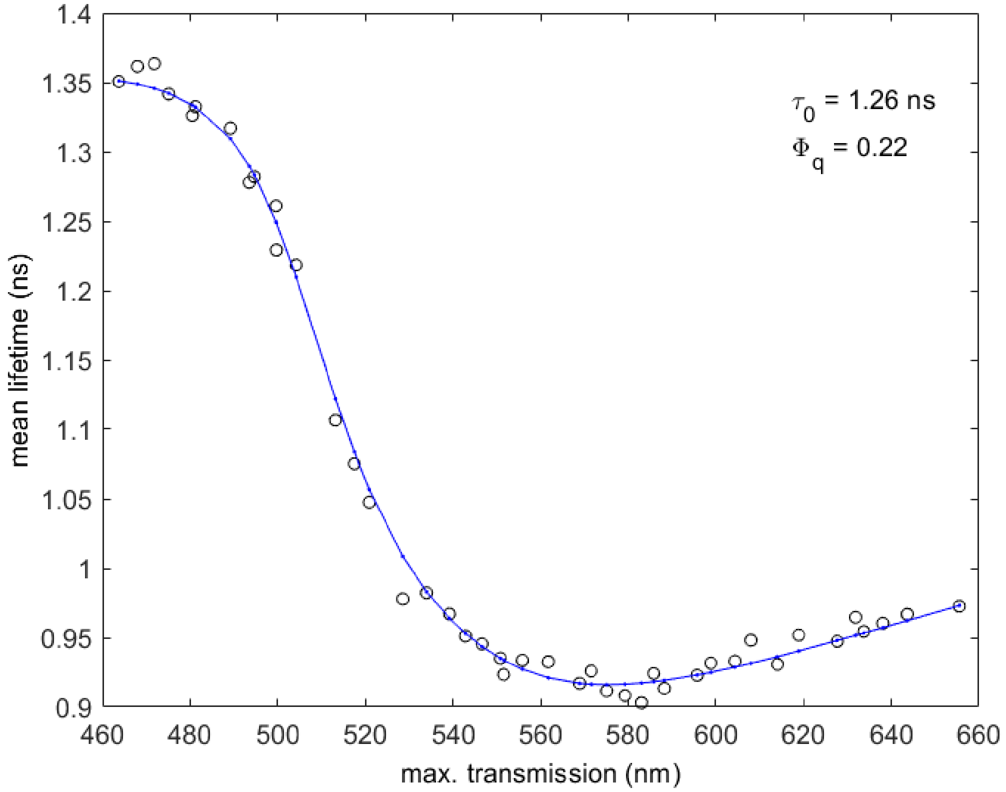

Supplementary Figure 18. (a) Excited state lifetime of DronpaM159T as a function of the maximum transmission wavelength (linearly proportional to the cavity length) of the nanocavity. The fit parameters are the fluorescence quantum yield (Φ) and the free lifetime in the absence of the cavity (τ).

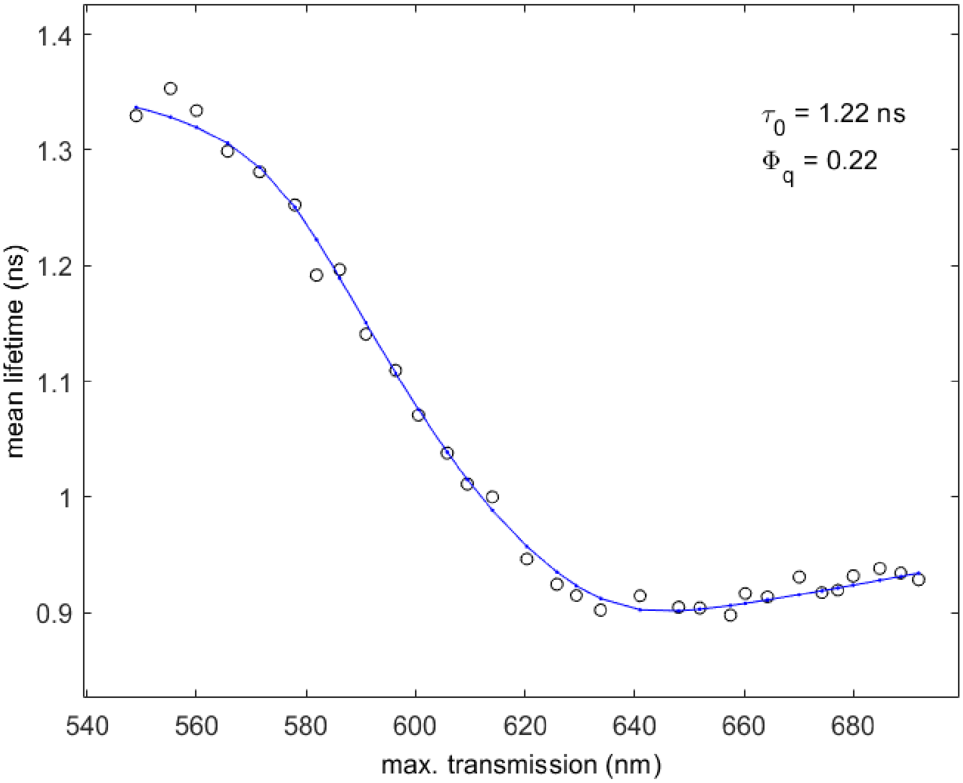

Supplementary Figure 19. (a) Excited state lifetime of rsCherry as a function of the maximum transmission wavelength (linearly proportional to the cavity length) of the nanocavity. The fit parameters are the fluorescence quantum yield (Φ) and the free lifetime in the absence of the cavity (τ).

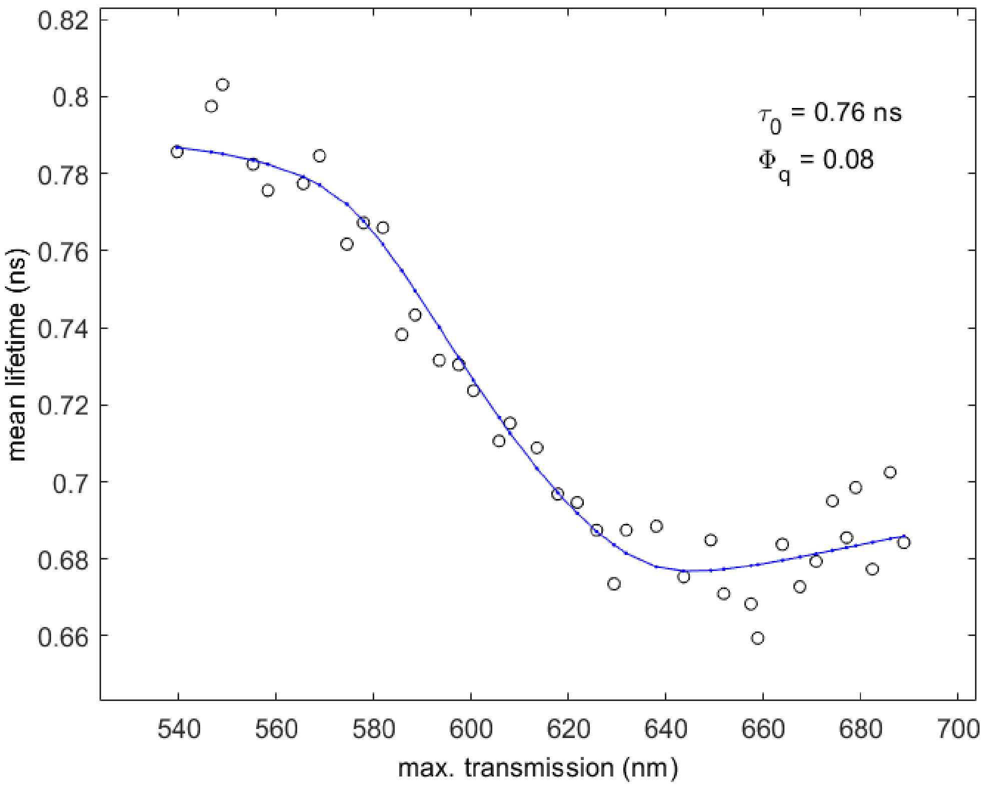

Supplementary Figure 20. (a) Excited state lifetime of rsCherryRev as a function of the maximum transmission wavelength (linearly proportional to the cavity length) of the nanocavity. The fit parameters are the fluorescence quantum yield (Φ) and the free lifetime in the absence of the cavity (τ).

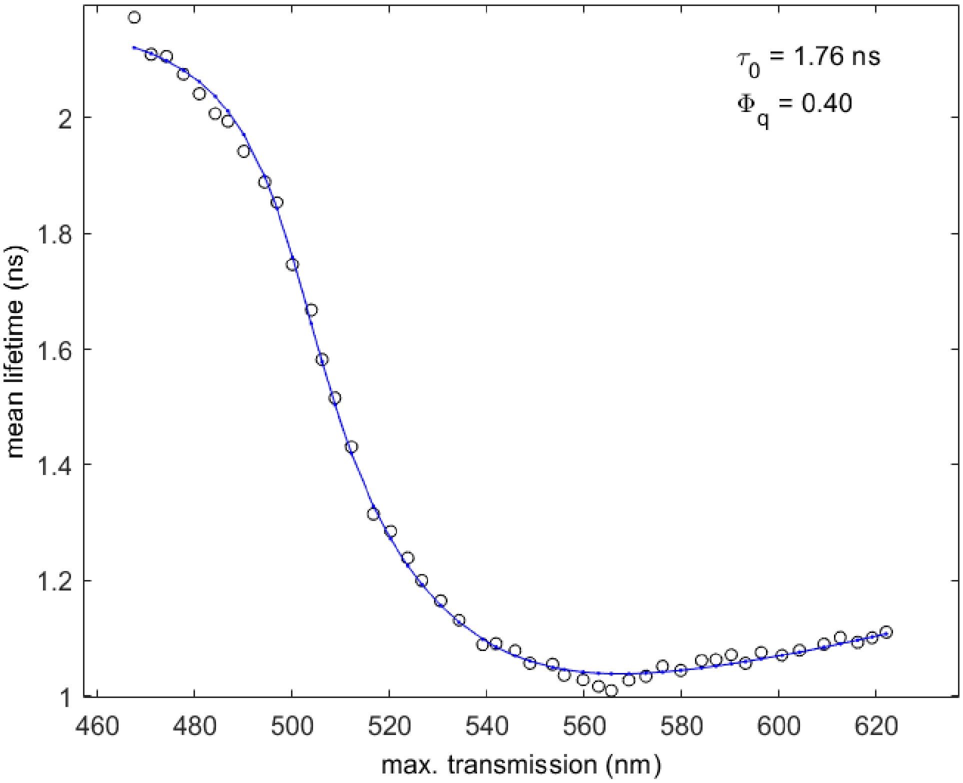

Supplementary Figure 21. (a) Excited state lifetime of rsEGFP as a function of the maximum transmission wavelength (linearly proportional to the cavity length) of the nanocavity. The fit parameters are the fluorescence quantum yield (Φ) and the free lifetime in the absence of the cavity (τ).

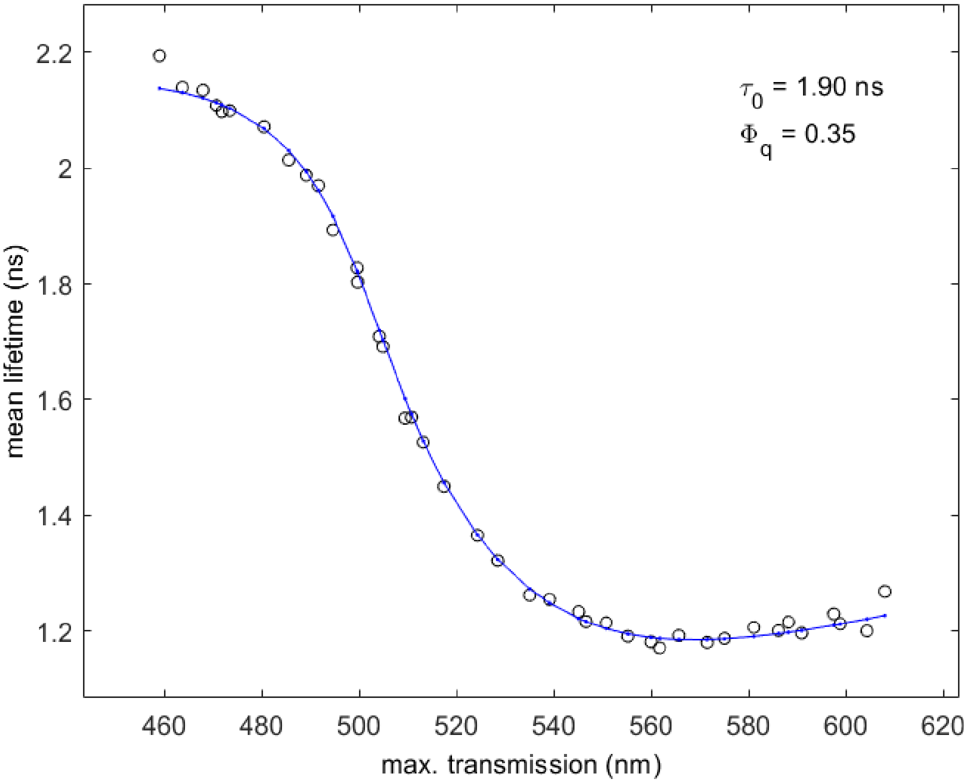

Supplementary Figure 22. (a) Excited state lifetime of rsEGFP2 as a function of the maximum transmission wavelength (linearly proportional to the cavity length) of the nanocavity. The fit parameters are the fluorescence quantum yield (Φ) and the free lifetime in the absence of the cavity (τ).

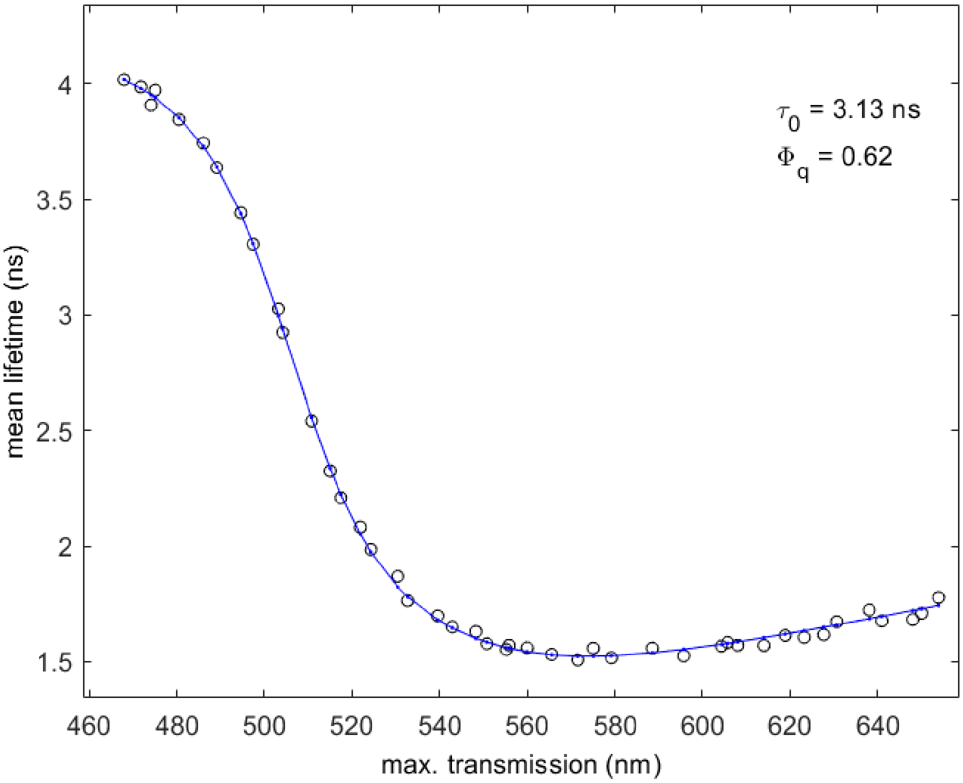

Supplementary Figure 23. (a) Excited state lifetime of rsFastLime as a function of the maximum transmission wavelength (linearly proportional to the cavity length) of the nanocavity. The fit parameters are the fluorescence quantum yield (Φ) and the free lifetime in the absence of the cavity (τ).

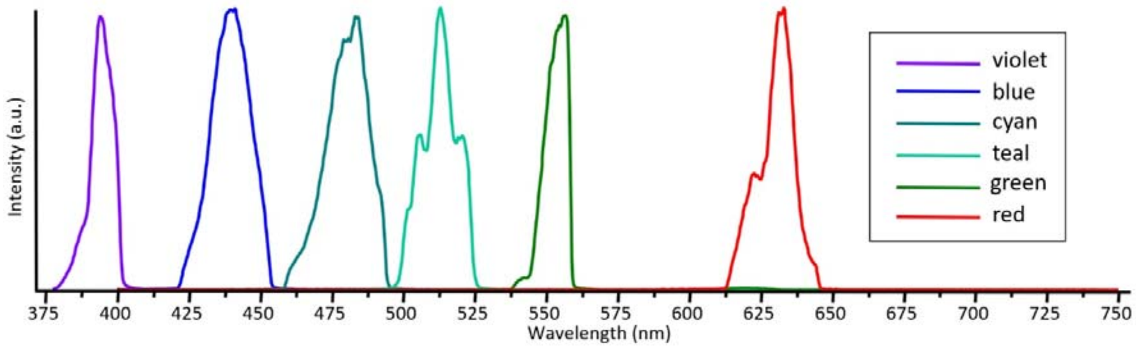

Supplementary Figure 24. Emission spectra of light emitting diodes.
